# Checkpoints modulation by the Human T-lymphotropic virus type 1 Tax protein

**DOI:** 10.1186/1742-4690-11-S1-P90

**Published:** 2014-01-07

**Authors:** Alexandre Carpentier, Pierre-Yves Barez, Mathieu Boxus, Luc Willems

**Affiliations:** 1 Molecular and Cellular Epigenetics, Interdisciplinary Cluster for Applied Genoproteomics (GIGA), University of Liège (ULg), Liège, Belgium; 2 Molecular Biology Lab, Gembloux Agro Bio-Tech (ULg), Gembloux, Belgium

## 

HTLV-1 is responsible for two main diseases, Adult T-cell Leukemia/Lymphoma and HTLV-1 Associated Myelopathy/Tropical Spastic Paraparesis, for which there is currently no satisfactory treatment. Among the proteins encoded by HTLV-1, Tax appears to play an important role in the mechanisms leading to pathogenicity. We are interested in the mechanisms of cell transformation by Tax and more particularly in the interplay between the viral Tax oncoprotein and the DNA damage response (DDR). We demonstrated that transient expression of Tax results in DNA damage, cell cycle arrest and activation of the DDR. In fibroblasts, cell cycle arrest occurs at the G1 and G2 phases depending on the p53 background. Although Tax induces apoptosis and senescence in fibroblasts, HTLV-1 infected lymphocytes proliferate continuously and appear to be adapted to the checkpoint control. This mechanism allows infected lymphocytes to proliferate despite the presence of genomic lesions. With these observations, we propose a novel therapeutic approach based on the principle of synthetic lethality.

